# Research on Aerodynamic Performance of Bionic Fan Blades with Microstructured Surface

**DOI:** 10.3390/biomimetics11010019

**Published:** 2025-12-31

**Authors:** Meihong Gao, Xiaomin Liu, Meihui Zhu, Chun Shen, Zhenjiang Wei, Zhengyang Wu, Chengchun Zhang

**Affiliations:** 1 Department of Mechanical and Electronic Engineering, Heze University, Heze 274015, China; gaomeihong@hezeu.edu.cn (M.G.); liuxiaomin@hezeu.edu.cn (X.L.); meihuizhu@hezeu.edu.cn (M.Z.); zhenjiangwei@hezeu.edu.cn (Z.W.); 2 Institute of International Education, New Era University College, Kajang 43000, Malaysia; 3 National Key Laboratory of Automotive Chassis Integration and Bionics, Jilin University, Changchun 130022, China; shench@jlu.edu.cn; 4 College of Mechanical & Electrical Engineering, Henan Agricultural University, Zhengzhou 450002, China; zywu24@henau.edu.cn

**Keywords:** bionic fan blades, microstructured surface, aerodynamic performance

## Abstract

The frictional resistance of impeller machinery blades such as aircraft engines, gas turbines, and wind turbines has a decisive impact on their efficiency and energy consumption. Inspired by the micro-tooth structure on the surface of shark skin, microstructural drag reduction technology has become a cutting-edge research direction for improving aerodynamic performance and a continuous focus of researchers over the past 20 years. However, the significant difficulty in fabricating microstructures on three-dimensional curved surfaces has led to the limited widespread application of this technology in engineering. Addressing the issue of drag reduction and efficiency improvement for small axial flow fans (local Reynolds number range: (36,327–40,330), this paper employs Design of Experiments (DOE) combined with high-precision numerical simulation to clarify the drag reduction law of bionic microgroove surfaces and determine the dimensions of bionic microstructures on fan blade surfaces. The steady-state calculation uses the standard k-ω model and simpleFoam solver, while the unsteady Large Eddy Simulation (LES) employs the pimpleFoam solver and WALE subgrid-scale model. The dimensionless height (h^+^) and width (s^+^) of microgrooves are in the range of 8.50–29.75, and the micro-grooved structure achieves effective drag reduction. The microstructured surface is fabricated on the suction surface of the blade via a spray coating process, and the dimensions of the microstructures are determined according to the drag reduction law of grooved flat plates. Aerodynamic performance tests indicate that the shaft power consumed by the bionic fan blades during the tests is significantly reduced. The maximum static pressure efficiency of the bionic fan with micro-dimples is increased by 2.33%, while that of the bionic fan with micro-grooves is increased by 3.46%. The fabrication method of the bionic microstructured surface proposed in this paper is expected to promote the engineering application of bionic drag reduction technology.

## 1. Introduction

The engine is the primary power device for fluid machinery. The fan system is the core component that ensures the operational performance of the engine. As the concept of low carbon has attracted widespread attention, drag reduction and efficiency enhancement have become one of the important design goals for fan blades. Research on the aerodynamic drag reduction performance of blades includes blade structure optimization and the application of surface microstructures. The fan blade already possesses an optimized low-drag aerodynamic shape. The approach of drag reduction through blade shape modification has reached a development plateau. The method of arranging microstructures on the blade surface remains capable of achieving a certain degree of drag reduction and further improve its aerodynamic efficiency [1,2,3,4].

After years of evolution, organisms in nature have developed biological structures that are adapted to drag reduction. For instance, the surface of the shortfin mako shark (*Isurus oxyrinchus*) is covered with dermal denticles. The studies have clarified the correlation between scales and the main flow field around the shark, providing a biological basis for bionic drag reduction design [5]. Bionic microstructure surfaces with drag reduction effects have been developed by studying the drag-reducing characteristics of shark skin [6,7,8]. Martin and Bhushan [9] found that shark-inspired microstructures can disrupt low-intensity, large-scale vortex structures, reducing turbulent kinetic energy by approximately 15–20% and achieving a drag reduction rate of up to 8%. Bai et al. [10] studied the drag reduction characteristics of textured surfaces and indicated that micro-textures reduced surface frictional resistance by 12–18%. The bionic microstructures on the surface of blades effectively reduce the surface frictional resistance and improve the fan’s aerodynamic performance [11,12,13]. Li et al. [14] found that the micro-grooves on the blade surface increased the pump’s efficiency by 2.7% and reduced drag by 5.3%. Wang et al. [15] developed mixed flow fans with bio-inspired grooves and achieved a 4.2% increase in static pressure efficiency while reducing shaft power consumption by 3.1%. Applying a grooved film to the surface of jet engine blades, the film not only provides oxidation protection at high temperatures (up to 600 °C), but also reduces surface frictional drag by 4.5–6.2% [16]. Compared with the smooth blade, the blade with micro-dimples improves aerodynamic efficiency by 3.8% and reduces drag by 9.1% [17]. Creating a slot in the blade can control the stall of centrifugal fan flow passage, and the slotted model can also control and mitigate boundary layer separation, significantly improving the blade’s aerodynamic performance [18]. The manufacturing technologies used to produce bionic non-smooth drag-reducing surfaces mainly include casting [19,20,21,22], laser etching [23,24,25,26], chemical vapor deposition [27], milling [28], grinding [29], photolithography [30,31,32], hot embossing [33,34,35], and 3D printing [36]. The development and application of bionic microstructure drag-reducing surfaces are still limited by processing technologies. As the scale of microstructures decreases, the manufacturing cost increases significantly. Therefore, low-cost and high-efficiency bionic microstructured drag-reducing surfaces are currently a research focus in the scientific community.

In this work, the size of microstructures that can reduce drag on the blade surface is determined according to the drag reduction law of bionic micro-grooved surfaces. A drag-reducing surface with micro-dimples and micro-grooves is fabricated on the suction side of a fan blade using a spray coating technique. Aerodynamic performance tests are conducted on both prototype and bionic fan blades in wind tunnel experiments. Compared with the prototype fan, the microstructured surface of the fan blade exerts a relatively small impact on the quantity of flow–static pressure curve. The bionic fan blade consumes less shaft power, which enhances the aerodynamic performance of the fan.

## 2. Bionic Design on the Surface of Fan Blades

To investigate the drag reduction law of bionic micro-grooved surfaces, a calculation scheme is designed using the DOE method. A quantitative analysis of drag reduction performance is achieved through high-precision numerical simulation methods. The standard k-ꞷ model and the simpleFoam steady-state solver are employed for steady-state calculations. For unsteady calculations, the results from the steady-state calculations are used as initial values. The pimpleFoam solver and the WALE subgrid-scale model are selected to implement a transient Large Eddy Simulation (LES).

### 2.1. Computational Domain and Boundary Conditions

**Figure 1:**
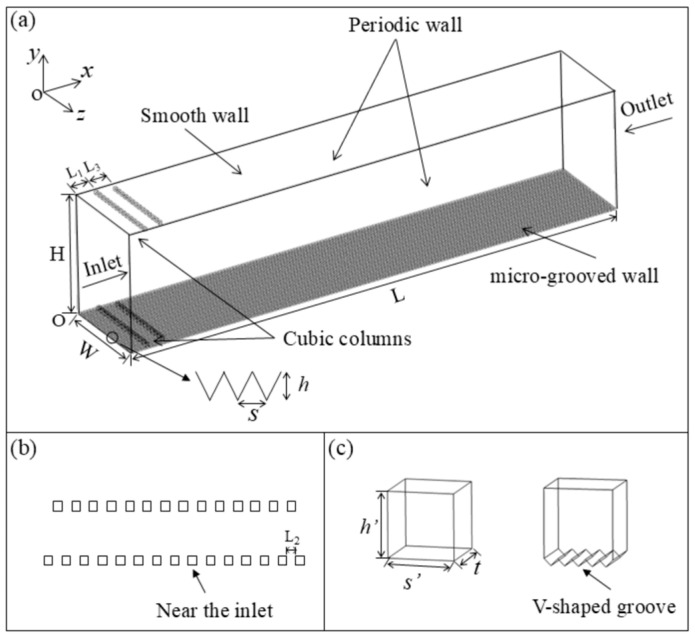
Computational domain and turbulence-generating cubic columns: (**a**) computational domain; (**b**) staggered cubic columns; (**c**) left: a cubic column with smooth wall, right: a cubic column with V-shaped groove wall.

The fluid domain of the bionic micro-grooved wall and the smooth wall is presented in Figure 1. The calculation domain is the channel with different upper and lower walls. The streamwise, normal, and spanwise directions are denoted by *x*, *y*, and *z*, respectively. A grooved plate is distributed along the *x* direction of the lower wall, and the upper wall is a smooth plate. The normal height of the computational domain is set to exceed 20 times the height of the V-shaped groove, while the streamwise length is specified to be more than 30 times the groove height. This configuration is designed to avoid the negative effects of boundary conditions on the flow fields of the upper and lower walls. The height and width of the V-shaped grooves are denoted by *h* and *s*, respectively. The streamwise length L is 85 mm, the normal height H is 20 mm, and the spanwise width W is 9.75 mm. Two rows of turbulence-generating cubic columns are designed on the upper and lower walls near the inlet to trigger early transition. The sizes of the cubic columns on the upper wall and the lower wall are the same, and the V-shaped cubic columns are embedded inside the surface of the groove on the lower wall. The length *s′*, width *t*, and height *h′* of single cubic column are 0.5 mm. The distance from the first row of cubic columns to the inlet (L_1_) is 3 mm, the spacing between two columns (L_2_) is 0.5 mm, the distance between the first row and the second row of the cubic column (L_3_) is 3 mm. The inlet corresponds to the given uniform velocity (5–100 m/s) and the outlet boundary condition is set as pressure. The upper and lower boundaries as well as the cubic columns correspond to the wall without slip. The boundary conditions of the periodic walls are set to be cyclicAMI.

### 2.2. Mesh Generation

**Figure 2:**
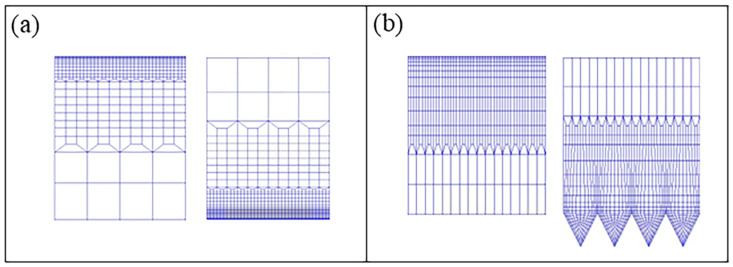
Details of the mesh near the wall: (**a**) smooth wall; (**b**) V-shaped groove wall.

The scale of the micro-grooved structure is extremely small, resulting in a very large number of meshes required for the computational flow field. Therefore, a layered refinement method is employed for meshing the computational model. The entire fluid domain is discretized into hexahedral meshes. In the *x* and *z* directions, the mesh is uniformly distributed, and the mesh dimensionless spacings are 7.72 and 1.64, respectively. A finer mesh must be generated in the near-wall region to accurately resolve the turbulent boundary layer. Non-uniform mesh is used in the *y* direction. The first layer dimensionless spacing is 0.78, which is sufficient to resolve the viscous sublayer of the flow. A Y-block division is adopted in the V-shaped groove. Figure 2 shows the grid layout near the V-shaped groove wall and smooth wall.

### 2.3. Grid Independence

The flow field of the grooved wall is simulated using the LES WALE subgrid-scale model at a local Reynolds number of 337,665. The drag of a grooved wall depends entirely on its surface frictional resistance. The surface friction coefficient *C_f_* for turbulent flow is given by Formula (1). The three sets of meshes with different densities are designed. The mesh refinement is mainly focused on the near-wall region and the V-shaped groove structure to ensure the resolution of the turbulent boundary layer. Systematically refined grid schemes with constant refinement ratios of $\sqrt{2}$ are applied to estimate the errors, as recommended by Wilson [37]. The surface friction coefficients of the grooved wall under three different grid schemes are compared and analyzed (Table 1). The relative error of the skin friction coefficient between Mesh 2 and Mesh 3 is only 0.186%, which demonstrates that Mesh 2 has sufficient density to accurately simulate the flow field around the micro-grooved structure.

(1) $$C_{f}=\frac{0.0742}{\sqrt[{5}]{{Re}_{x}}}$$

(2) Rex=ρUxμ

*Re_x_* is the local Reynolds number, *x* is the distance from the inlet, *ρ* is the density of air, and μ is the dynamic viscosity.

### 2.4. Drag Reduction Law of Bionic Micro-Grooved Surfaces

**Figure 3:**
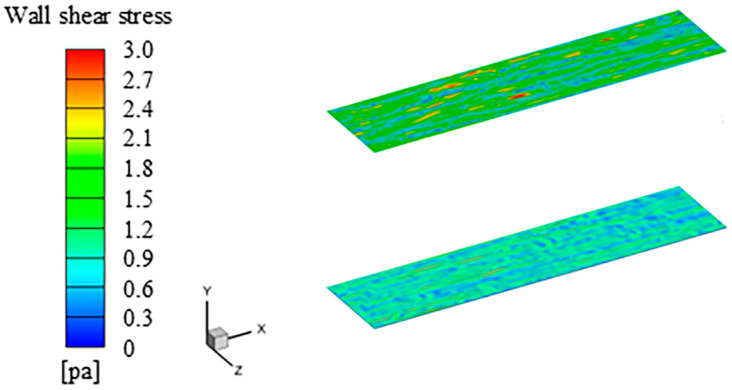
The wall shear stress of the smooth surface and the grooved surface. The upper is the smooth surface, and the lower is the grooved surface.

Figure 3 shows the wall shear stress of the smooth surface and the grooved surface at an incoming flow velocity of 20 m/s. The instantaneous drag exhibits periodic variation characteristics, and the drag reduction rate is calculated based on the time-averaged drag. *D_s_* and *D_r_* denote the instantaneous friction resistance of the smooth wall and the groove wall, respectively. *A_s_* and *A_r_* represent the areas of the smooth wall and the groove wall, respectively. *DR* is the rate of drag reduction, and ${\bar{D}}_{s}$ and ${\bar{D}}_{r}$ represent the time-averaged resistance values of the smooth surface and the groove surface, respectively. Wall shear stress of the grooved surface and the smooth surface are 4.5 × 10^−4^ pa and 4.8 × 10^−4^ pa, respectively. This indicates that the grooves exert a drag reduction effect under turbulent flow conditions, with a drag reduction rate of 6.95%.

(3) $$D_{s}=\mu \int_{A_{s}}\frac{\partial u}{\partial y}dA_{s}$$

(4) $$D_{r}=\mu \int_{A_{r}}\frac{\partial u}{\partial y}dA_{r}$$

(5) $$DR(\%)=\frac{{\bar{D}}_{s}-{\bar{D}}_{r}}{{\bar{D}}_{s}}\times 100$$

Table 2 shows the drag reduction rates of the micro-grooved wall under different microstructural sizes and the friction Reynolds numbers. It can be concluded that the dimensionless height and width of the grooves are in the ranges of 8.50 ≤ *h^+^* ≤ 29.75 and 8.50 ≤ *s^+^* ≤ 29.75, and the grooved structure is drag-reducing. The dimensionless height *h*^+^ and dimensionless width *s*^+^ can be given by the following formula:

(6) $$h^{+}=h\frac{u_{\tau }}{\nu }$$

(7) $$s^{+}=s\frac{u_{\tau }}{\nu }$$

(8) Reτ=δuτν

(9) $$\delta =0.381\frac{x}{\sqrt[{5}]{Re_{x}}}$$

Here, $u_{\tau }$ is the wall friction velocity, ν is kinematic viscosity, ${Re}_{\tau }$ is the frictional Reynolds number, and δ is the thickness of the turbulent boundary layer.

### 2.5. Determination of the Size of Bionic Microstructures on the Surface of the Fan Blade

The scales of the shortfin mako shark (*Isurus oxyrinchus*) have a longitudinal groove structure on the surface [5], while the cross-section of individual scales presents a micro-dimpled shape [36]. The two structural features can reduce the frictional resistance of the shark’s body during swimming. The conclusions of the bionic groove drag reduction inspired by shark skin are taken as the theoretical basis. The fan blade is approximately considered a flat plate. The size of the blade’s microstructure with a drag reduction effect is determined based on the flow field parameters of the blade.

**Figure 4:**
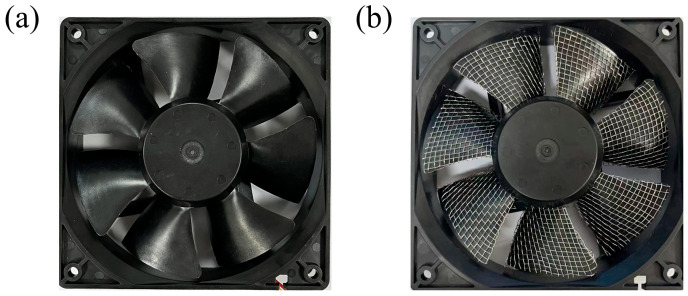
The fan blades: (**a**) prototype fan blades; (**b**) fan blades covered with stainless steel mesh.

Figure 4 shows the fan blade for testing. The voltages are 7.6 v (3630 rpm), 8.0 v (3760 rpm), 8.4 v (3890 rpm), and 8.8 v (4030 rpm) in the experiment. The flow velocity of the blade is calculated using Formula (10). The suction surface of the fan blade with microstructures is set as a rotating wall, and the rotational angular velocity is calculated using Formula (11).

(10) v=ω×r

(11) $$\omega =\frac{2\pi n}{60}$$

Here, ω is the rotational angular velocity of the fan blade, *r* = 41.25 mm is the radius of the fan blade, and *n* is the rotational speed of the fan blade.

Table 3 shows the microstructure sizes of the blade surface at various voltages. The height and width ranges of the microstructures on the fan blade surface are 0.080 mm ≤ *h* ≤ 0.304 mm and 0.080 mm ≤ *s* ≤ 0.304 mm. Stainless steel mesh with a diameter of 0.30 mm is selected, and it is completely and tightly attached to the suction surface of the fan blade (Figure 4b).

## 3. Bionic Microstructures on Blade Surfaces

### 3.1. Fabrication of Microstructured Surfaces

**Figure 5:**
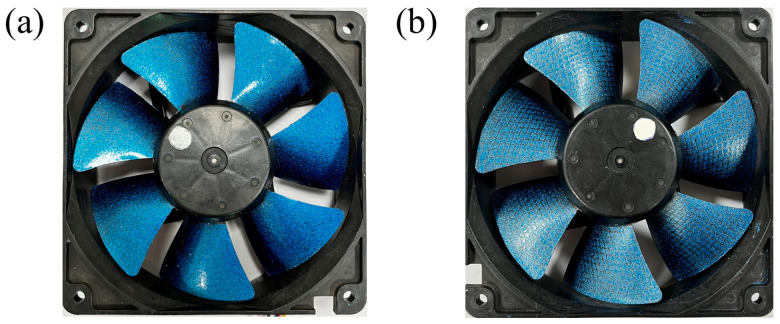
The bionic fan blade: (**a**) fan blades with micro-dimples; (**b**) fan blades with micro-grooves.

Table 4 shows the components of the coating, sequence of configuration, and mass percentage. β-type phthalocyanine blue exhibits high crystallinity, wear resistance, stability, and strong tinting strength. It does not undergo chemical changes even at a temperature of 500 °C. The scaly structure of non-floating aluminum powder can reduce the penetration of oxygen, water vapor, and other corrosive substances. The surface of the aluminum powder slurry is coated with a layer of unsaturated fatty acid, which deposits in the middle and lower layers of the coating film under the action of gravity. Polydimethylsiloxane resin enhances the surface tension gradient within the coating, which favors the formation of surface dimples. Thus, the fabrication of surfaces featuring microstructural dimples involves the addition of polydimethylsiloxane resin. No polydimethylsiloxane is added to the coating for micro-grooves, and the mass percentage of FEVE fluorocarbon resin is increased to 67%. The bionic fan blade is presented in Figure 5.

### 3.2. Morphological Characteristics of Bionic Microstructured Surfaces

**Figure 6:**
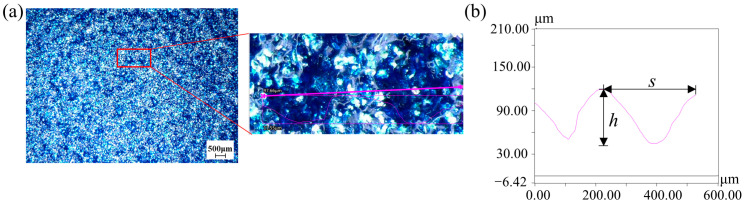
The dimpled morphology and geometric parameters of the bionic fan: (**a**) dimpled surface and an enlarged region; (**b**) cross-sectional curve of the dimples (dimple depth *h*, dimple width *s*).

**Figure 7:**
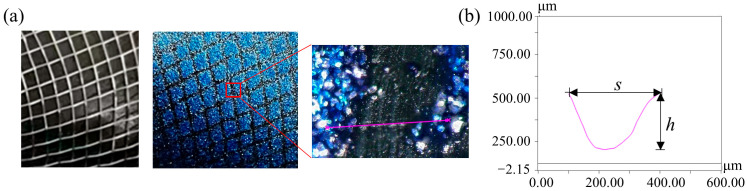
The grooved morphology and geometric parameters of the bionic fan: (**a**) grooved surface and an enlarged region; (**b**) cross-sectional curve of the grooves.

The morphology and size of the bionic microstructural dimples and grooves on the blade surface were measured using ultra-depth-of-field measuring equipment (VHX-6000, Keyence, Japan). The maximum resolution of the equipment is 1.3 nm and supporting 1600 × 1200 pixels. The maximum frame rate is 50 F/s. Figure 6 shows the surface of the bionic fan blade with dimple-shaped microstructures and the dimples are approximately circular or elliptical. During the curing process of the coating film, the volatilization of the solvent causes the temperature of the outer surface to drop rapidly, while the temperature inside the coating film system drops slowly. This leads to a higher surface tension on the outer layer of the coating film and a lower surface tension inside the system. It is precisely the surface tension gradient caused by the temperature gradient that drives the material in the coating film system to continuously migrate from the center of the vortex cell unit to the edge. This migration results in the depression of the vortex cell center and the bulging of the edge, and the micro-dimple structure is formed. To minimize errors arising from local variations in microstructure morphology, ten regions with areas of 1 mm^2^ were randomly selected in each blade to obtain the mean density (number of micro-dimples, mm^−2^). The mean density of the micro-dimples is 6 ± 2 mm^−2^. The depth *h* of the dimple ranges from 0.089 mm to 0.105 mm, and the width *s* is from 0.119 mm to 0.298 mm. Figure 7 presents the surface of the bionic fan blade with grooved microstructures. Both the height and width of the grooves are 0.30 mm. The fabricated micro-grooves exhibit a periodic structure with a spacing of 0.60 mm. The stainless steel mesh is aligned parallel to the streamwise direction of the blade’s suction surface and the alignment is consistent with the dominant flow direction of the blade surface.

## 4. Experimental Investigation of the Aerodynamic Performance of Bionic Fan Blades

### 4.1. Methods

**Figure 8:**
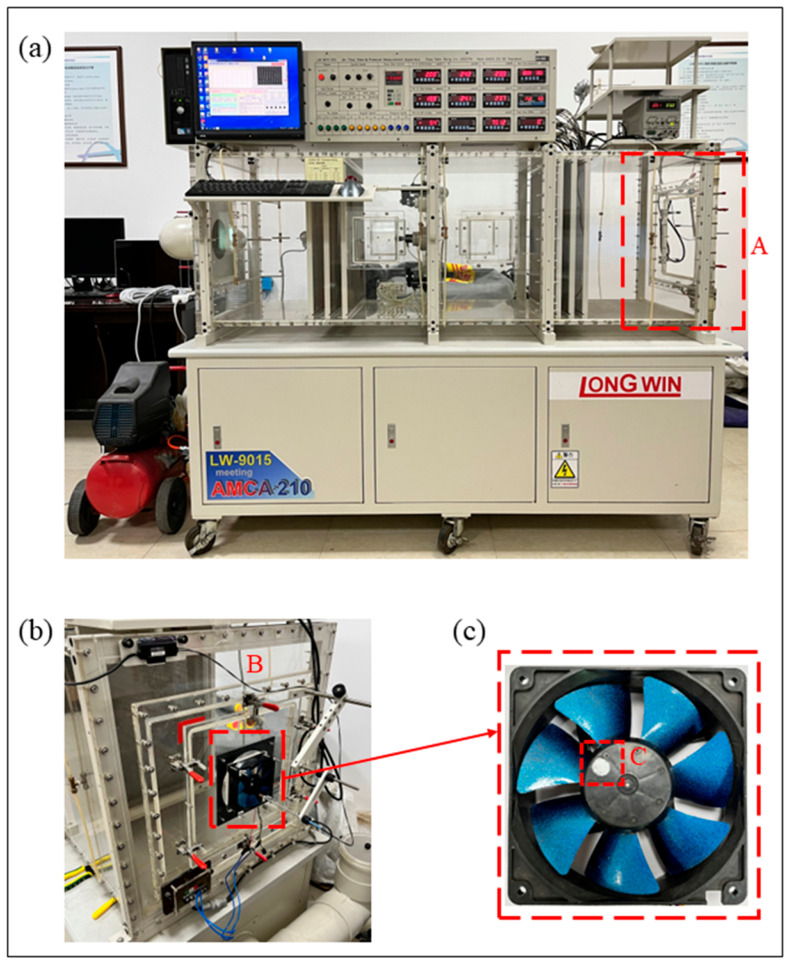
The fully automatic air flow and pressure measuring equipment and tested fan: (**a**) the measuring equipment; (**b**) the installation position of the fan; (**c**) the tested fan. Region A: the position of the wind tunnel end plate. Region B: the fan installation position. Point C: the reflective sticker.

The aerodynamic performance of the prototype and bionic fan blades was tested using the fully automatic air flow and pressure measuring equipment (LW-9015-250, Taiwan, China) shown in Figure 8a. Region A denotes the position of the wind tunnel end plate. The flow rate range of the equipment is 2.4–250 CFM. The static pressure range is 0–120 mmAq. The accuracy of voltage and current is ±0.5%, and the accuracy of rotational speed is ±1.0%. The maximum diameter of the tested fan is 140 mm. The constant voltage mode was adopted in the fan test. The tested fan installed at the wind tunnel port is shown in Figure 8b. Region B is the fan installation position. Figure 8c shows the tested bionic fan. A laser is aligned with the reflective sticker (test point C) to measure the rotational speed of the fan blades. The distance between the laser and the reflective sticker is about 20 cm. The aerodynamic performance of the prototype and bionic fan blades was tested at different rotational speeds, under the condition of a stable external wind field. The fully automatic air flow and pressure measurement wind tunnel is controlled by a fully automated computer system, which enables the acquisition of the flow rate–static pressure curve, flow rate–shaft power curve, and flow rate–static pressure efficiency curve of the fan blade. The static pressure efficiency (*η_es_*) of the fan is determined by the effective static pressure power (*N_e_*) and the input shaft power *N*.

(12) $$\eta_{es}=N_{es}/N$$

(13) $$N_{es}=QP_{s}/1000$$

Here, *Q* is the flow rate and *P_s_* denotes the static pressure.

### 4.2. Results and Discussion

#### 4.2.1. Quantity of Flow–Static Pressure Curve

**Figure 9:**
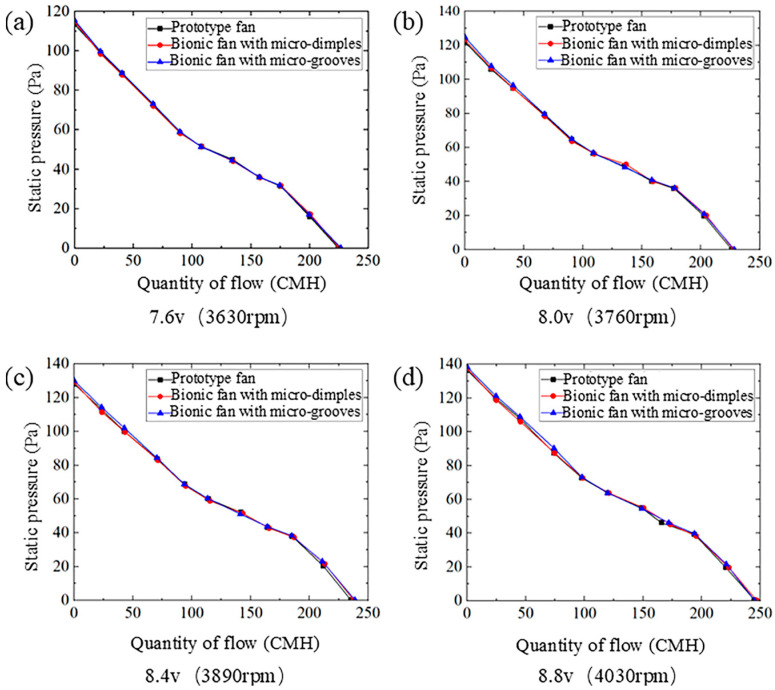
Quantity of flow–static pressure curves for prototype and bionic fans: (**a**) voltage 7.6 v, rotational speed 3630 rpm; (**b**) voltage 8.0 v, rotational speed 3760 rpm; (**c**) voltage 8.4 v, rotational speed 3890 rpm; (**d**) voltage 8.8 v, rotational speed 4030 rpm.

Figure 9 shows the quantity of flow–static pressure curves of the prototype fan and the bionic fans under four different rotational speeds. The quantity of flow–static pressure curves for the prototype and bionic fans exhibit the same trend. As the quantity of flow increases, the static pressure decreases gradually. There are slight differences in the maximum static pressure and maximum flow rate between the prototype fan and the bionic fans. The compared values between the prototype fan and the bionic fan are shown in Table 5. At a rotational speed of 3630 rpm, the maximum static pressure of the bionic fan with micro-dimples increases by 1.23% compared to the prototype fan. At 3760 rpm, the maximum increase in maximum static pressure of the bionic fan with micro-grooves compared to the prototype fan is 2.36%. Under the rotational speeds of 3890 rpm and 4030 rpm, the increases in maximum flow rate of the bionic fan with micro-dimples relative to the prototype fan are quite similar, at 0.89% and 0.90%, respectively. The bionic fan with micro-grooves exhibits a maximum flow rate enhancement of 1.41% over the prototype fan. The two bionic structures on the blade surface exert a relatively small influence on the quantity of flow–static pressure curve of the fan.

#### 4.2.2. Quantity of Flow–Shaft Power Curves

**Figure 10:**
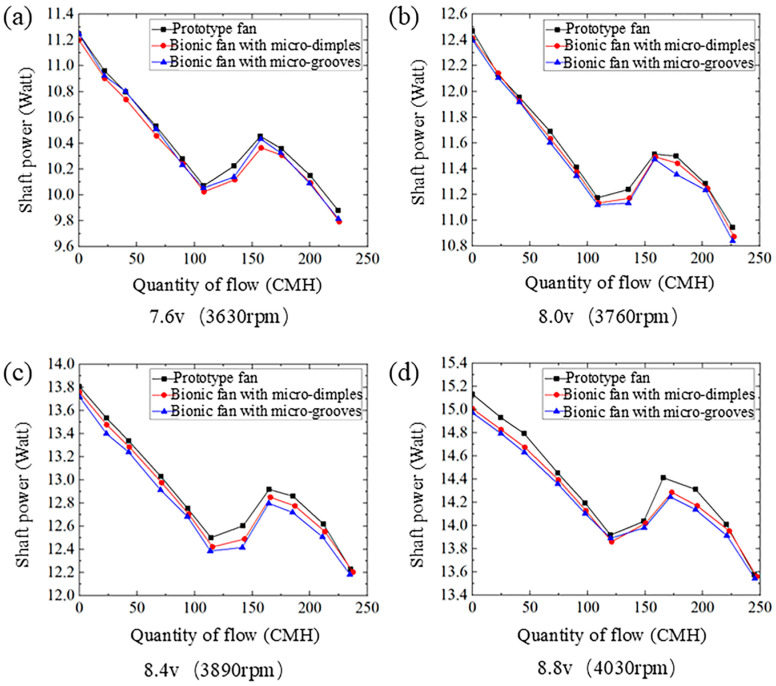
The quantity of flow–shaft power curves of the prototype and the bionic fans: (**a**) voltage 7.6 v, rotational speed 3630 rpm; (**b**) voltage 8.0 v, rotational speed 3760 rpm; (**c**) voltage 8.4 v, rotational speed 3890 rpm; (**d**) voltage 8.8 v, rotational speed 4030 rpm.

Figure 10 shows the quantity of flow–shaft power curves of the prototype and the bionic fans under four different rotational speeds. The quantity of flow–shaft power curve exhibits an initial decrease, followed by a rise and a subsequent decrease. Both bionic fans require less shaft power than the prototype. The shaft power consumed by the fan increases as the rotational speed rises. Table 6 lists the shaft power at the maximum flow rate for both the prototype and bionic fans. At a rotational speed of 3630 rpm, the bionic fan with micro-dimples consumes the lowest shaft power compared to the prototype fan. Its relative value is −0.92%. Under the rotational speed of 3760 rpm, a 1.01% reduction in shaft power consumption is observed for the micro-grooved bionic fan relative to the prototype fan.

#### 4.2.3. Quantity of Flow–Static Pressure Efficiency Curves

**Figure 11:**
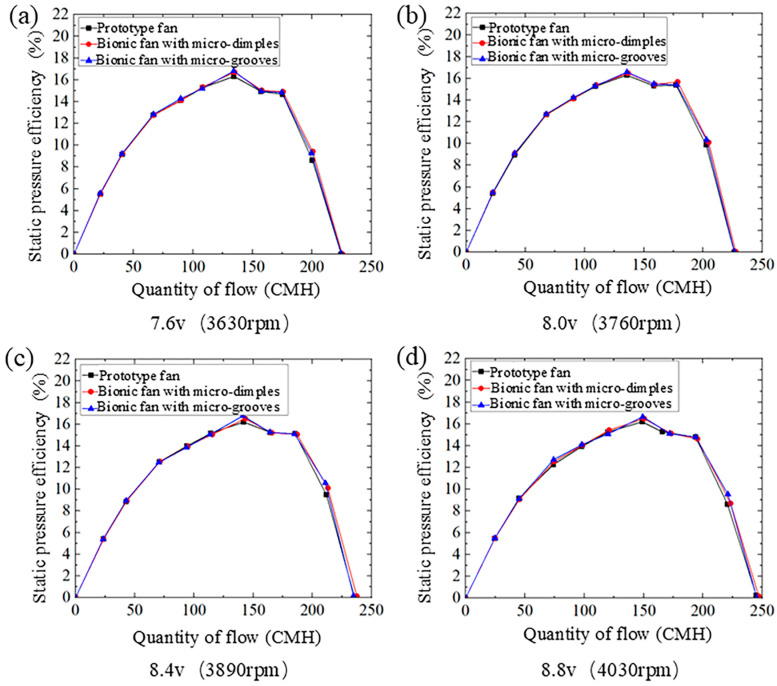
The quantity of flow–static pressure efficiency curves of the prototype and the bionic fans: (**a**) voltage 7.6 v, rotational speed 3630 rpm; (**b**) voltage 8.0 v, rotational speed 3760 rpm; (**c**) voltage 8.4 v, rotational speed 3890 rpm; (**d**) voltage 8.8 v, rotational speed 4030 rpm.

Figure 11 shows the quantity of flow–static pressure efficiency curves of the prototype fan and the bionic fans under four different rotational speeds. With increasing flow rate, the static pressure efficiency follows a trend of an initial increase, followed by a decrease, a minor recovery, and a final decline to zero. There is a maximum point of static pressure efficiency within the flow rate. Table 7 presents the maximum static pressure efficiency, error, and efficiency improvement for both the prototype and bionic fans. At a rotational speed of 3630 rpm and a flow rate of 134 CMH, the bionic fan with dimples achieves the largest increase in maximum static pressure efficiency, and its relative value is 2.33%. The maximum static pressure efficiency of the bionic fan with micro-grooves reached its highest increase of approximately 3.46% at a rotational speed of 3890 rpm and a flow rate of 148 CMH. The maximum static pressure efficiency of the bionic fans is higher than that of the prototype fan, which indicates that the application of dimple or groove structures on the blade surface enhances aerodynamic efficiency.

## 5. Conclusions

The scales on a shark’s body are characterized by a longitudinal groove structure. The cross-section of an individual scale resembles the curve of a dimple. The rapid and efficient fabrication of on-demand controllable micro-dimpled or micro-grooved surfaces on the suction side of fan blades is achieved based on the structural features of shark scales. For microstructural dimensions ranging from 0.080 mm to 0.304 mm in both height and spacing, the bionic fan blade may enhance aerodynamic efficiency, thereby enabling drag reduction. The spraying process was used to fabricate micro-dimples with a depth of 0.089–0.105 mm and a width of 0.119–0.298 mm, and micro-grooves with both height and width of 0.30 mm. A wind tunnel study was carried out to assess the aerodynamic performance of both the prototype and bionic fan blades. The presence of micro-dimple and micro-groove structures had a minor effect on the flow–static pressure curve of the fan. Compared to the prototype fan, the bionic fan blades consumed less shaft power and achieved higher maximum static pressure efficiency. This indicates that the microstructures of the surface disrupt low-intensity vortex structures and reduce turbulent kinetic energy. Under a rotational speed of 3630 rpm, the bionic fan with micro-dimples achieved a 2.33% relative improvement in maximum static pressure efficiency. The maximum static pressure efficiency of the micro-grooved bionic fan increased by 3.46% at 3890 rpm. This improvement in static pressure efficiency is consistent with the performance of bionic micro-textured surfaces reported in centrifugal pumps [14] and aero-engine blades [16], confirming that bionic microstructures can effectively optimize the aerodynamic performance of small axial flow fans. At 3760 rpm, the micro-grooved bionic fan exhibits the lowest shaft power consumption, with a relative value of 1.01% compared to the prototype fan. The microstructures on the blade surface enhance the aerodynamic performance of the fan, reducing drag and improving efficiency. In the future, the combination of macro blade shape optimization and microstructural surfaces can be used to achieve synergistic drag reduction and efficiency improvement.

## Figures and Tables

**Table 1:** Study of grid independence.

Mesh Set	Total Number of Elements	Surface Friction Coefficient	Relative Error vs. Mesh 3 (%)
Mesh 1 (Coarse)	15,707,459	0.005983	1.371
Mesh 2 (Medium)	22,005,849	0.005912	0.186
Mesh 3 (Fine)	30,800,186	0.005901	**-**

**Table 2:** The drag reduction rate of the micro-grooved wall.

	*h* (mm)	*h^+^*	*s* (mm)	*s^+^*	*U* (m/s)	*Re_τ_*	${\bar{D}}_{s}$ (N)	${\bar{D}}_{r}$ (N)	*DR* (%)
1	0.101	8.58	0.101	8.58	24.3	258	0.00065	0.00061	6.15
2	0.249	21.16	0.101	8.58	24.3	258	0.00064	0.00059	7.81
3	0.101	8.58	0.249	21.16	24.3	258	0.00108	0.00106	1.85
4	0.249	21.16	0.249	21.16	24.3	258	0.00111	0.00107	3.60
5	0.101	25.29	0.101	25.29	80.7	599	0.00511	0.00448	12.33
6	0.249	62.34	0.101	25.29	80.7	599	0.00519	0.00546	−5.20
7	0.101	25.29	0.249	62.34	80.7	599	0.00784	0.00882	−12.5
8	0.249	62.34	0.249	62.34	80.7	599	0.00830	0.01058	−27.47
9	0.050	8.50	0.175	29.75	52.5	443	0.00276	0.00270	2.17
10	0.300	51	0.175	29.75	52.5	443	0.00280	0.00308	−10.00
11	0.175	29.75	0.050	8.50	52.5	443	0.00187	0.00181	3.21
12	0.175	29.75	0.300	51	52.5	443	0.00322	0.00359	−11.49
13	0.175	3.58	0.175	3.58	5	85	0.0000334	0.0000342	−2.39
14	0.175	53.14	0.175	53.14	100	695	0.00800	0.00962	−20.25
15	0.175	29.75	0.175	29.75	52.5	443	0.00270	0.00268	0.72
16	0.175	29.75	0.175	29.75	52.5	443	0.00269	0.00266	0.79
17	0.175	29.75	0.175	29.75	52.5	443	0.00271	0.00268	0.74
18	0.175	29.75	0.175	29.75	52.5	443	0.00264	0.00261	0.75
19	0.175	29.75	0.175	29.75	52.5	443	0.00261	0.00259	0.76
20	0.175	29.75	0.175	29.75	52.5	443	0.00265	0.00262	0.75
21	0.175	29.75	0.175	29.75	52.5	443	0.00268	0.00265	0.78
22	0.175	29.75	0.175	29.75	52.5	443	0.00260	0.00258	0.77
23	0.175	29.75	0.175	29.75	52.5	443	0.00266	0.00263	0.73

The height *h* (0.05 mm–0.3 mm), the width *s* (0.05 mm–0.3 mm), flow velocity *U* (5 m/s–100 m/s). A positive value of the drag reduction rate indicates drag reduction, while a negative value indicates drag increase.

**Table 3:** The microstructure sizes of the blade surface.

Voltage (v)	*N* (rpm)	*Re_x_*	*u_τ_* (m/s)	*h* (mm)		*s* (mm)	
				*h*_min_	*h*_max_	*s*_min_	*s*_max_
7.6	3630	36,327	1.458	0.088	0.304	0.088	0.304
8.0	3760	37,628	1.505	0.085	0.295	0.085	0.295
8.4	3890	38,929	1.552	0.083	0.286	0.083	0.286
8.8	4030	40,330	1.603	0.080	0.277	0.080	0.277

**Table 4:** The components of the coating, sequence of configuration, and mass percentage.

Components of the Coating	Sequence of Configuration	Mass Percentage
Toluene	1	3.0
Ethyl acetate	2	10.0
β-type phthalocyanine blue	3	3.0
Non-floating aluminum powder	4	3.0
FEVE fluorocarbon resin	5	66.5
Polydimethylsiloxane resin	6	0.5
Xylene	7	4.0
Hexamethylene diisocyanate	8	10.0

**Table 5:** Comparison of maximum static pressure and maximum flow rate between the prototype and bionic fans.

Voltage/Rotational Speed	Comparison of Maximum Static Pressure (%)		Comparison of Maximum Flow Rate (%)	
	Dimples Versus Prototype	Grooves Versus Prototype	Dimples Versus Prototype	Grooves Versus Prototype
7.6 (3630)	1.23	1.80	0.35	0.91
8.0 (3760)	1.03	2.36	0.54	0.92
8.4 (3890)	0.71	1.45	0.89	1.41
8.8 (4030)	0.75	1.43	0.90	0.19

**Table 6:** Shaft power and relative variation at the maximum flow rate for both the prototype and bionic fans.

Voltage/Rotational Speed	Shaft Power (Watt)			Comparison of Shaft Power (%)	
	Prototype	Dimple	Groove	Dimple Versus Prototype	Groove Versus Prototype
7.6 (3630)	9.88	9.79	9.81	−0.92	−0.71
8.0 (3760)	10.94	10.87	10.83	−0.64	−1.01
8.4 (3890)	12.23	12.20	12.18	−0.25	−0.41
8.8 (4030)	13.57	13.56	13.54	−0.07	−0.22

**Table 7:** Comparison of maximum static pressure efficiency between the prototype and bionic fans.

Voltage/Rotational Speed	Fan Type	Maximum Static Pressure Efficiency (%)	Error (±%)	Efficiency Improvement (%)
7.6 (3630)	prototype	16.31	±0.5	-
	micro-dimple	16.70	±0.3	2.33
	micro-groove	16.81	±0.4	2.97
8.0 (3760)	prototype	16.30	±0.4	-
	micro-dimple	16.49	±0.5	1.15
	micro-groove	16.58	±0.5	1.69
8.4 (3890)	prototype	16.20	±0.3	-
	micro-dimple	16.51	±0.6	1.88
	micro-groove	16.78	±0.4	3.46
8.8 (4030)	prototype	16.19	±0.5	-
	micro-dimple	16.49	±0.6	1.82
	micro-groove	16.65	±0.4	2.76

## Data Availability

The original contributions presented in this study are included in the article material. Further inquiries can be directed to the corresponding author.

## References

[1] Büttner C.C., Schulz U. (2011). Shark skin inspired riblet coatings for aerodynamically optimized high temperature applications in aeroengines. Adv. Eng. Mater..

[2] Liu Y., Yuan Q. (2024). Optimizing Bionic Blades for Multi-blade Centrifugal Fans: Asymmetric Thickness Inspired by Carangiform Fish. J. Appl. Fluid Mech..

[3] Zhu Q., Zhang C., Yu F.H., Xu Y. (2024). Investigation on drag reduction on rotating blade surfaces with microtextures. Beilstein J. Nanotechnol..

[4] Zhang W., Yu D.H., Li G.C., Zhao C.Y., Liu Z.N. (2025). The mechanisms of drag reduction through bionic microstructures on fan blade surfaces. Phys. Fluids.

[5] Zhang C.C., Gao M.H., Liu G.Y., Zheng Y.H., Xue C., Shen C. (2022). Relationship Between Skin Scales and the Main Flow Field Around the Shortfin Mako Shark *Isurus oxyrinchus*. Front. Bioeng. Biotechnol..

[6] Zhang K.S., Li J., Zhang K.Z., Zhang J. (2025). Optimization of surface microgrooves and their performance and mechanism of synergistic drag reduction with bionic mucus. Ocean. Eng..

[7] Wang Y.M., Lu C.J., Cui C.H., Lu W.J., Sun J.Y., Fan J.J., Zhang Y.F. (2025). A review of research on drag reduction and energy-saving of non-smooth biomimetic structure and its application. Mater. Today Commun..

[8] Heidarian A., Ghassemi H., Liu P. (2018). Numerical analysis of the effects of riblets on drag reduction of a flat plate. J. Appl. Fluid Mech..

[9] Martin S., Bhushan B. (2014). Fluid flow analysis of a shark-inspired microstructure. J. Fluid Mech..

[10] Bai Q., Bai J., Meng X. (2016). Drag reduction characteristics and flow field analysis of textured surface. Friction.

[11] Wang S., Rong R., Wu Z.R., Zhang L. (2013). Research on drag-reduction characteristic of non-smooth surface riblet structure on aerofoil blade of centrifugal fan. Zhongguo Dianji Gongcheng Xuebao/Proc. Chin. Soc. Electr. Eng..

[12] Wu Z.R., Hao X.F., Rong R., Wang S.L. (2014). Study on drag-reduction mechanism of riblet surface on aerofoil blade of centrifugal fan. Xitong Fangzhen Xuebao/J. Syst. Simul..

[13] Wu Z.R., Hao X.F., Rong R., Wang S.L. (2014). Three-dimensional numerical analysis on drag reduction characteristics of the riblet structure for centrifugal fan blades. Zhongguo Dianji Gongcheng Xuebao/Proc. Chin. Soc. Electr. Eng..

[14] Li Q.Y., Hu Y.H., Chen J., Jiang H., Ning T.Z. (2025). Effect of biomimetic microstructure on blade surface on drag reduction performance of centrifugal pump. J. Mech. Electr. Eng..

[15] Wang J., Nakata T., Liu H. (2019). Development of Mixed Flow Fans with Bio-Inspired Grooves. Biomimetics.

[16] Büttner C.C., Schulz U. (2011). Shark skin inspired riblet structures as aerodynamically optimized high temperature coatings for blades of aeroengines. Smart Mater. Struct..

[17] Zhang C., Saurav B.K. (2018). Investigation on drag reduction performance of aero engine blade with micro-texture. Aerosp. Sci. Technol..

[18] Rong R., Cui K., Li Z.J., Wu Z.R. (2015). Numerical Study of Centrifugal Fan with Slots in Blade Surface. Procedia Eng..

[19] Zhao D.Y., Huang Z.P., Wang M.J. (2012). Vacuum casting replication of micro-riblets on shark skin for drag-reducing applications. J. Mater. Process. Technol..

[20] Chen H., Che D., Zhang X. (2015). Large-proportional shrunken bio-replication of shark skin based on UV-curing shrinkage. J. Micromechanics Microengineering.

[21] Liu Y., Li G. (2012). A new method for producing “Lotus Effect” on a biomimetic shark skin. J. Colloid Interface Sci..

[22] Luo Y., Zhang D., Liu Y. (2015). Chemical, Mechanical and Hydrodynamic Properties Research on Composite Drag Reduction Surface Based on Biological Sharkskin Morphology and Mucus Nanolong Chain. J. Mech. Med. Biol..

[23] Bixler G.D., Bhushan B. (2013). Shark skin inspired low-drag microstructured surfaces in closed channel flow. J. Colloid Interface Sci..

[24] Bixler G.D., Bhushan B. (2013). Bioinspired micro/nanostructured surfaces for oil drag reduction in closed channel flow. Soft Matter.

[25] Kietzig A.M., Lehr J., Matus L. Laser-induced patterns on metals and polymers for biomimetic surface engineering. Laser Applications in Microelectronic and Optoelectronic Manufacturing (LAMOM) XIX. Proceedings of the SPIE LASE.

[26] Solomon B.R., Khalil K.S., Varanasi K.K. (2014). Drag reduction using lubricant-impregnated surfaces in viscous laminar flow. Langmuir.

[27] Fischer A.C., Belova L.M., Rikers Y.G.M. (2012). 3D Free-form patterning of silicon by ion implantation, silicon deposition, and selective silicon etching. Adv. Funct. Mater..

[28] Barbier C., Jenner E., D’Urso B. (2014). Large Drag Reduction over Superhydrophobic Riblets. arXiv.

[29] Denkena B., Köhler J., Wang B. (2010). Manufacturing of functional riblet structures by profile grinding. CIRP J. Manuf. Sci. Technol..

[30] Bixler G.D., Bhushan B. (2012). Bioinspired rice leaf and butterfly wing surface structures combining shark skin and lotus effects. Soft Matter.

[31] Siegel F. (2009). Extensive Micro-Structuring of Metals using Picosecond Pulses—Ablation Behavior and Industrial Relevance. J. Laser Micro/Nanoeng..

[32] Henoch C., Krupenkin T.N., Kolodner P. Turbulent drag reduction using superhydrophobic surfaces. Proceedings of the 3rd AIAA Flow Control Conference.

[33] Han X., Zhang D.Y., Li X. (2008). Bio-replicated forming of the biomimetic drag-reducing surfaces in large area based on shark skin. Chin. Sci. Bull..

[34] Choi K.S. (2013). Smart flow control with riblets. Adv. Mater. Res..

[35] Lu B., Meng W.J. (2014). Roll Molding of Microchannel Arrays on Al and Cu Sheet Metals: A Method for High-Throughput Manufacturing. J. Micro Nano-Manuf..

[36] Wen L., Weaver J.C., Lauder G.V. (2014). Biomimetic shark skin: Design, fabrication and hydrodynamic function. J. Exp. Biol..

[37] Wilson R.V., Stern F., Coleman H.W., Paterson E.G. (2011). Comprehensive approach to verification and validation of CFD simulations—Part 2: Application for rans simulation of a cargo/container ship. J. Fluids Eng. Trans. ASME.

